# Expression of mutant CHMP2B linked to neurodegeneration in humans disrupts circadian rhythms in *Drosophila*

**DOI:** 10.1096/fba.2019-00042

**Published:** 2019-07-11

**Authors:** DaWon Lee, Xiaoyue Zheng, Kay Shigemori, Christopher Krasniak, Jie Bin Liu, Chao Tang, Joshua Kavaler, S. Tariq Ahmad

**Affiliations:** ^1^ Department of Biology Colby College Waterville Maine; ^2^ Present address: Industrial Economics, Inc. 2067 Massachusetts Ave. Cambridge MA 02140; ^3^ Present address: Cold Spring Harbor Laboratory 1 Bungtown Road Cold Spring Harbor NY 11724; ^4^ Present address: Dana‐Farber Cancer Institute 450 Brookline Ave. Boston MA 02215; ^5^ Present address: McIntyre School of Commerce, University of Virginia Charlottesville VA 22904

**Keywords:** CHMP2B, circadian Rhythms, endosomal Lysosomal Pathway, ESCRT

## Abstract

Mutations in CHMP2B, an ESCRT‐III (endosomal sorting complexes required for transport) component, are associated with frontotemporal dementia (FTD) and amyotrophic lateral sclerosis (ALS). Neurodegenerative disorders including FTD are also associated with a disruption in circadian rhythms, but the mechanism underlying this defect is not well understood. Here, we ectopically expressed the human CHMP2B variant associated with FTD (CHMP2B^Intron5^) in flies using the *GMR*‐GAL4 driver (*GMR*>CHMP2B^Intron5^) and analyzed their circadian rhythms at behavioral, cellular, and biochemical level. In *GMR*>CHMP2B^Intron5^ flies, we observed disrupted eclosion rhythms, shortened free‐running circadian locomotor period, and reduced levels of *timeless* (*tim*) mRNA—a circadian pacemaker gene. We also observed that the *GMR*‐GAL4 driver, primarily known for its expression in the retina, drives expression in a subset of *tim* expressing neurons in the optic lobe of the brain. The patterning of these *GMR*‐ and *tim*‐positive neurons in the optic lobe, which appears distinct from the putative clusters of circadian pacemaker neurons in the fly brain, was disrupted in *GMR*>CHMP2B^Intron5^ flies. These results demonstrate that CHMP2B^Intron5^ can disrupt the normal function of the circadian clock in *Drosophila*.

## 1 INTRODUCTION

The endosomal‐lysosmal pathway delivers transmembrane proteins to the lysosomal lumen for degradation.^1^, ^2^, ^3^ CHMP2B, a subunit of Endosomal Sorting Complex Required for Transport‐III (ESCRT‐III), mediates the endosomal sorting of ubiquitinated transmembrane proteins into the multivesicular bodies (MVBs) for transport to lysosomes.^4^, ^5^ Consequently, CHMP2B and other ESCRT subunits play a critical role in the regulation of multiple cell surface receptors and their signaling cascades.^1^, ^2^ CHMP2B also contributes in the autophagy pathway—a critical and highly regulated process for protein and organelle homeostasis, by regulating fusion of the autophagosome with the lysosome.^6^ Additionally, CHMP2B has been shown to perform neuron‐specific roles during the formation and maintenance of synapses.^7^ Multiple studies have suggested that disruption of ESCRT function may be associated with a number of diseases including cancer, pathogenic infections, and neurodegenerative diseases such as FTD3, ALS, Huntington's disease, paraparesis, and prion disease.^2^, ^8^, ^9^ However, it remains poorly understood how a mutant protein such as the mutant isoform of CHMP2B associated with frontotemporal dementia (CHMP2B^Intron5^) leads to neurodegeneration. One mechanism attributed to the CHMP2B^Intron5^‐mediated defects is misregulation of receptor‐signaling pathways.

We have previously characterized the effects of CHMP2B^Intron5^ on receptor‐mediated signaling in *Drosophila*. We have shown that ectopic expression causes melanotic deposits in the eye due to upregulation of the Toll receptor and its signaling.^10^ We have also demonstrated that expression of CHMP2B^Intron5^ causes upregulation of the Notch receptor and its signaling resulting in defects in cell fate determination during early stages of eye development^11^ and during development of sensory bristles on the notum (Wilson et al unpublished). These studies indicate that CHMP2B^Intron5^ differentially affects physiological processes in a cellular and developmental context‐dependent manner.

Neurodegenerative disorders, such as FTD are also known to cause disruptions in the sleep/wake circadian rhythm physiology.^12^, ^13^ Circadian rhythms rely on three main components: a central pacemaking clock, sensory input which can reset the clock, and physiological output in response to the clock. The central clock itself is governed by the oscillations of protein levels of circadian pacemaker genes (eg, *tim, period [per], clock [clk], cycle [cyc], cryptochrome [cry]*). The main source of sensory input is light, which can reset the clock by modulating circadian pacemaker protein levels in central and peripheral clock cells (eg, in the retina and ocelli). The central clock signals broadly throughout the organism, controlling physiological processes such as locomotion and eclosion.^14^, ^15^, ^16^

Light entrainment of circadian rhythms is achieved by photopigments such as Cry in the ventral lateral neurons (LN_v_s) in central clock cells and opsins in peripheral clocks cells (eyes, ocelli, and Hofbauer‐Buchner (H‐B) eyelet).^15^

The central clock acts as the circadian rhythm pacemaker and is composed of approximately 150 pacemaker neurons in the *Drosophila* brain. The pacemaker neurons are divided into several subgroups: dorsal neuron groups 1‐3 (DN1, DN2, DN3), dorsal lateral neurons (LN_D_), lateral posterior neurons, large lateral ventral neurons (l‐LN_v_), and small lateral ventral neurons (s‐LN_v_s).^14^, ^15^ These pacemaker neurons express circadian pacemaker genes in overlapping and partially differential patterns.^14^ At the molecular level, the central clock is regulated by cyclic feedback loops of synthesis and degradation of the circadian pacemaker proteins.^15^, ^17^

One of the best characterized behavioral outputs of circadian rhythm, which allows for high throughput monitoring, is the cycling of locomotor activity. Lack of locomotion correlates with the sleep phase, whereas bouts of locomotion reflects the wake phase. The normal locomotor activity pattern during a 24‐hour period is crepuscular in *Drosophila* that is, active during dawn and dusk. *Drosophila* maintained in a 12 hour light‐12 hour dark (LD) cycle show two peaks of locomotor activity around light on‐off (M‐peak) and around light off‐on (E‐peak) transitions. These two activity peaks are present even when the animal is maintained in a 24 hour dark (DD) cycle. The time interval between daily onset of bouts of locomotor activity when an animal is maintained in a DD cycle is known as free‐running locomotor period. The s‐LN_v_ neurons are necessary and sufficient to generate the M‐peak and free‐running locomotor period.^18^, ^19^ Analysis of the free‐running locomotor period allows for characterization of underlying molecular genetic pathways without the overbearing influence of light to reset the expression and degradation cycles of circadian pacemaker proteins.^15^, ^17^

Another physiological output of circadian rhythm is the timing of eclosion of adults from pupae. Since the eclosion event happens only once in the lifetime of a fly, eclosion rhythms are monitored at the population level. Wild type flies preferentially eclose around dawn possibly because in the wild the air is most moist around dawn and therefore prevents dessication of freshly exposed fragile cuticle.^20^ Eclosion rhythms are regulated by coordination between central clock cells and peripheral clock cells. This coordination is achieved via the release of a neuropeptide (pigment dispersing factor) by the LN_v_s.^21^

To uncover the effects of CHMP2B^Intron5^ on circadian locomotor and eclosion rhythms, we used the GAL4‐UAS system to ectopically express CHMP2B^Intron5^ in *Drosophila* under the control of the GMR‐GAL4 driver which is primarily known to drive transgene expression in cells posterior to the morphogenetic furrow of larval eye discs.^22^ However, other studies have suggested that the *GMR*‐GAL4 driver has a broader expression profile outside the eye imaginal disc, including in the wing imaginal discs, leg discs, trachea, brain,^23^ and the neuronal cells of the cerebral ganglia.^24^ The *GMR*‐positive cells in adult eyes contribute in photic input for circadian light entrainment. This approach allows us to infer how CHMP2B^Intron5^ or a dysfunctional endosomal‐lysosomal pathway can manifest into deficits in circadian rhythms.

## 2 MATERIALS AND METHODS

### 2.1 Fly stocks

*Drosophila* were raised on a standard Nutri‐Fly^TM^ Bloomington Formulation (Genesee Scientific) at 25°C under 12 hour light‐12 hour dark (LD) cycle, unless otherwise stated. *Canton‐S*, *w^1118^*, UAS‐GFPnls (stock # 4776), and UAS‐RedStinger (stock # 8547) stocks were obtained from the Bloomington *Drosophila* Stock Center. The UAS‐Hid (EP‐Hid—EP(3)30060) was generously provided by Eric Lai. The *GMR*>CHMP2B^Intron5^ line was previously generated by recombining *GMR*‐GAL4 (Bloomington stock #8605) and UAS‐CHMP2B^Intron5^ transgenic constructs on the second chromosome.^10^ *GMR*‐GAL4 was crossed with *w^1118^* to generate *w; GMR*‐GAL4/+ flies which were used as control in free‐running circadian rhythm assay.

A *timeless* (*tim*) reporter line (*tim*‐DsRed) was generated as follows. A region that spanned −1231 to +2799 relative to the *tim* transcription start site was amplified by PCR from *CantonS* genomic DNA using Q5 High Fidelity DNA polymerase (New England Biolabs) and cloned into the BamHI site of pRed H‐stinger.^25^ The primers used to generate the amplicon were 5′‐GCGGATCCGCTGAAGTGG and 5′‐ TTGTGGATCCGCCTAACTCTGC. Several *tim*‐DsRed transgenic lines were produced through injections of the created plasmid and the helper plasmid pUChsΔ2‐3 into embryos using standard methods.^26^ A single representative line was selected for this study. This line closely recapitulates the expression pattern observed with a previously generated *tim*‐GAL4 line (Bloomington stock #7126, unpublished observations).

### 2.2 Circadian locomotor and eclosion rhythms

For the behavioral assays, free‐running circadian locomotor periods of *w^1118^*, *GMR*‐GAL4/+, and *GMR*>CHMP2B^Intron5^ flies were calculated using the *Drosophila* Activity Monitor (DAM; Trikinetics) system as per protocols previously described.^27^ Briefly, the flies were first exposed to 12 hour light‐12 hour dark (LD) cycle for 3 days for entrainment to light followed by 5‐7 days of 24 hours dark (DD) to calculate the free‐running period. Activity counts collected in 10‐minute bins during DD phase were used to determine entrainment and calculate free‐running period (Clock Lab).

The composite actograms were created by averaging the activity of all flies of a given genotype of a given trial at each 10‐minute bin (Clock Lab). The composite actograms were used to generate representative images of the locomotor rhythm. Flies with robust morning and evening activity peaks with consistent period of approximately 24 hours duration were considered entrained. The free‐running periods of different genotypes were tested for statistical significance using ANOVA and post‐hoc Bonferroni test for pairwise comparison in R statistical programming language.

Eclosion rhythms were determined by placing the pupae of appropriate genotype in the *Drosophila* activity monitors using the same setup as described above. The timing of eclosion was determined by the initiation of locomotor activity of individual adult flies as they emerge from the pupal case in 12 hour light‐12 hour dark (LD) cycle. The data was obtained in 1‐hour bins. The difference in eclosion preference during the morning peak between genotypes was compared by the chi‐squared test.

### 2.3 Development and life span assay

The development rate was documented for *Canton‐S* (as wild type control) and *GMR*>CHMP2B^Intron5^ strains. Seven vials containing 100 *Canton‐S* larvae each and five vials containing 100 *GMR*>CHMP2B^Intron5^ larvae each were raised under the standard conditions. For every vial, the number of pupae and adult development were documented daily. To preserve the natural development from pupa to fly, the pupae were not disturbed, and their count was recorded by directly marking the location of pupa on the vial. The study continued until flies stopped emerging. The development rate is reported as percentages of pupa and adult flies in each vial. The development rate was tested for statistical significance using Welch's *t* test in the R statistical programming language.

The survival rate was determined for *Canton‐S* and *GMR*>CHMP2B^Intron5^ strains. Ten vials containing ten 1‐2‐day‐old adult flies each and five vials containing 8‐10 adult 1‐2‐day‐old *GMR*>CHMP2B^Intron5^ flies were raised in standard conditions. For each vial, the number of viable flies was recorded daily for 65 days by which point all flies had perished. To avoid counting progeny, surviving larvae were transferred to new vials whenever they have emerged from food for pupation. The survival rate is reported as percentage of viable flies.

### 2.4 In situ fluorescence analysis

For the whole‐brain fluorescent imaging, adult brains were dissected, including the removal of retina and lens tissue, and fixed with 4% paraformaldehyde in phosphate‐buffered saline (PBS) for 1 hour at room temperature. Brains were washed six times with PBS plus 0.2% TritonX (PBX). Brains for antibody labeling were incubated in a solution of PBX with 2% bovine serum albumin (BSA) containing a 1:100 dilution of monoclonal anti‐Flag primary antibody (Sigma‐Aldrich) overnight at 4°C. The brains were then washed six times with PBX and incubated with a 1:500 dilution of AlexaFluor 488 goat anti‐mouse (Invitrogen, Eugene, OR) in PBX with 2% BSA overnight at 4°C. After a final six washes with PBX, brains were mounted in EMS Shield Mount with DABCO mounting medium (Electron Microscopy Sciences, Hatfield, PA) and imaged using an AxioCam MRm camera on an Imager A2 fluorescent microscope (Zeiss, Thornwood, NY).

The disruption of patterning in the band of *GMR*‐positive cells in optic lobe by expressing CHMP2B^Intron5^ and Hid was quantified by cropping the area of the optic lobe images of these flies to only include the central section of the optic lobe which contained this band of expression. The images were then thresholded such that any pixel with fluorescence levels 1.5 standard deviations above the mean of the cropped image was assigned a value of 1, and all others a value of 0. Finally, the thresholded image was fitted to a quadratic formula of the form ax^2 + bx + c to calculate the R^2^ value as an estimate of goodness of fit. The higher the R^2^ value, the more band‐like the expression pattern and the healthier the GMR expressing cells. Image analysis was done using custom software written in MATLAB Release 2018a using the Image Analysis Toolbox. Independent images from different fly preparations for each genotype (n = 5) was averaged and was tested for statistical significance using Welch's t test in the R statistical programming language.

### 2.5 Quantitative Real‐Time PCR

Total RNA from 25 fly heads of appropriate genotype was extracted at the zeitgeber time ZT6 and ZT12 (ie, 6 hours and 12 hours after lights on for flies maintained in 12 hour light‐12 hour dark (LD) cycle) using TRIzol reagent (Invitrogen) according to the manufacturer's protocol. Total RNA was converted to cDNA and amplified with specific primers and SYBR Green using One‐Step RT‐PCR kit (Applied Biosystems) on an ABI7700 sequence detection system (Applied Biosystems). A standard curve was generated for each reaction set. Expression was normalized to *RP49* transcript values and calculated with the Delta‐Delta‐Ct method. The transcript levels were tested for statistical significance using Welch's *t* test and two‐way ANOVA in R statistical programming language. Primers for *tim*:qRT‐PCR: Forward primer: 5′CCCTTATACCCGAGGTGGAT3′; Reverse primer: 5′TGATCGAGTTGCAGTGCTTC3′. Primers for RP49 qRT‐PCR: Forward primer: 5′CGGTTACGGATCGAACAAGC3′.

Reverse primer: 5’CTTGCGCTTCTTGGAGGAGA3’.

## 3 RESULTS

### 3.1 *GMR*‐driven CHMP2B^Intron5^ expression causes circadian locomotor rhythm deficits

**Figure 1:**
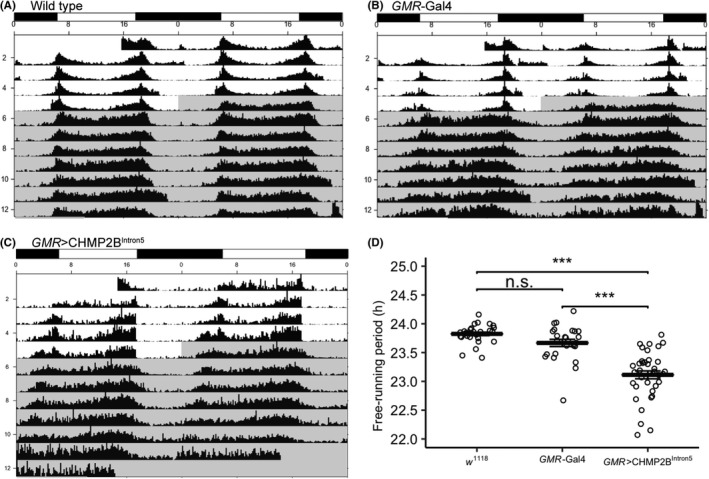
Composite double‐plotted actograms of 1‐3‐day‐old (A) wild type (*w^1118^*), (B) *GMR*‐GAL4, (C) *GMR*>CHMP2B^Intron5^ flies, and (D) a scatter plot of the free‐running locomotor period of the three genotypes show that *GMR*‐driven expression of CHMP2B^Intron5^ causes a significant reduction in the free‐running locomotor rhythm (wild type—23.82 ± 0.03 hours (n = 29); *GMR*‐GAL4—23.67 ± 0.06 hours (n = 25); *GMR*>CHMP2B^Intron5^—23.11 ± 0.07 hours (n = 37); error bars—standard error of measurement; n.s.—not significant; ***—*P* < 0.001; ANOVA, Bonferroni pairwise comparison). The timing of 12:12 LD and DD cycles is shown by alternating black and white rectangles on top of the actograms and by light gray shading on the actograms, respectively

Wild type (*w^1118^*) and *GMR*‐GAL4 flies showed good light entrainment in the 12 hour light‐12 hour dark (LD) cycle and normal free‐running locomotor rhythms in the DD cycle (Figure 1). The *w^1118^* fly strain was used as a wild type control because *GMR*‐GAL4 and UAS‐CHMP2B^Intron5^ transgenic lines were made in the *w^1118^* background. The free‐running period of wild type and *GMR*‐GAL4 flies was 23.82 ± 0.03 hours (n = 29) and 23.67 ± 0.06 hours (n = 25), respectively (Figure 1A,B). In *GMR*>CHMP2B^Intron5^ flies the light entrainment appeared normal as shown by consistent daily timing of onset and offset of locomotor activity in the LD cycle (Figure 1C). However, during the DD cycle, *GMR*>CHMP2B^Intron5^ flies showed a significantly reduced free‐running period (23.11 ± 0.07 hours, n = 37, *P* < 0.001, ANOVA; Bonferroni pairwise comparison) (Figure 1C). The shortening of the free‐running period did not show an age‐dependent decline between 1‐3‐day‐old and 3‐week‐old *GMR*>CHMP2B^Intron5^ flies (Supplementary Figure S1).

To further characterize the roles of *GMR*‐positive cells and CHMP2B^Intron5^ in circadian rhythms, we selectively ablated *GMR*‐positive cells. For this, we used the *GMR*‐GAL4 driver to express Head involution defective (Hid)—a well‐characterized apoptosis effector (*GMR*>Hid). *GMR*>Hid flies showed severe circadian rhythm deficits manifested as lack of light entrainment of locomotor rhythms in 12 hour light‐12 hour dark (LD) cycle and except for a modest evening peak had no discernable rhythm in both 12:12 LD and DD cycles (Supplementary Figure S2). Taken together, CHMP2B^Intron5^ disrupts free‐running circadian locomotor rhythms, and *GMR* driver expression pattern appears to include cells that modulate circadian rhythms.

### 3.2 *GMR*‐driven CHMP2B^Intron5^ expression affects eclosion rhythms but does not affect eclosion rate and longevity

**Figure 2:**
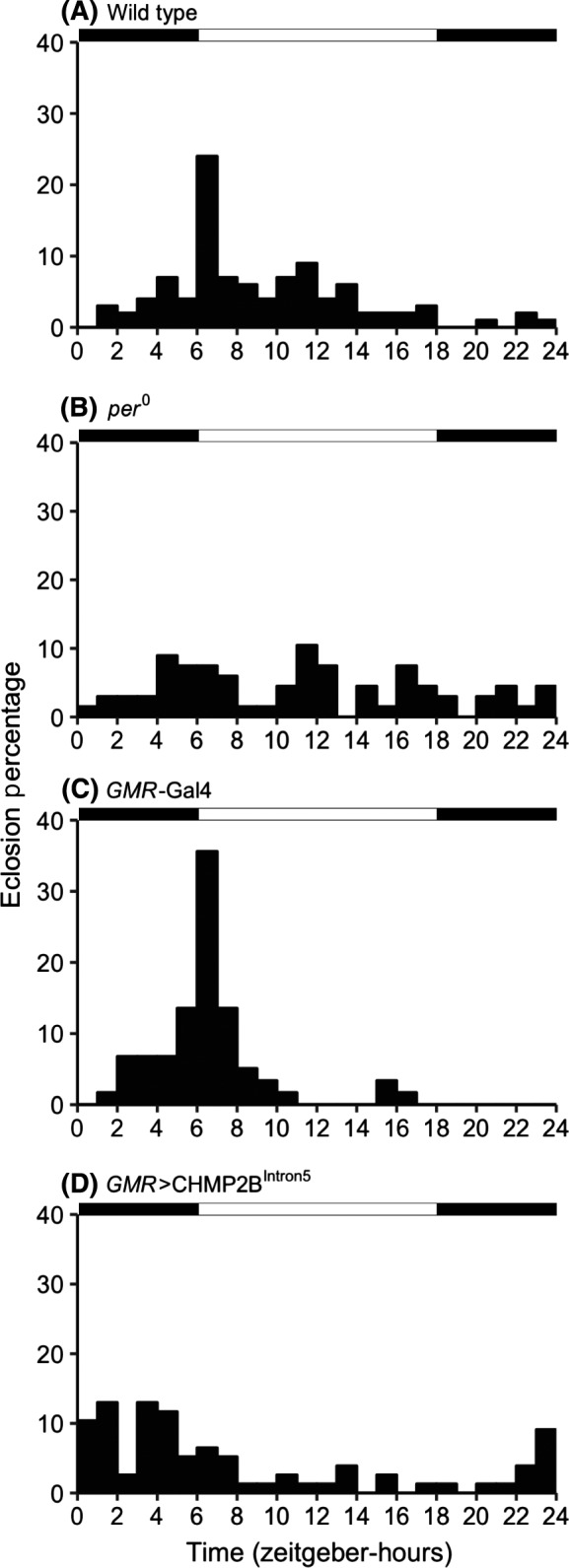
Histograms plotting the eclosion percentage in 1 hour bins of (A) wild type (*Canton‐S*), (B) *per^0^*, (C) *GMR*‐GAL4, and (D) *GMR*>CHMP2B^Intron5^ flies maintained in 12 hour light‐12 hour dark (LD) cycle. The wild type and *GMR*‐GAL4 flies preferentially eclose around light on‐off transition (n_wt_ = 100, n*_GMR_*_‐Gal4_ = 59). The *per^0^* flies lack robust circadian preference for eclosion (*P* < 0.01; chi‐squared test; n*_per0_* = 67). The *GMR*>CHMP2B^Intron5^ flies show a significant dampening of the eclosion peak around light on‐off transition (*P* < 0.01; chi‐squared test; n = 77). The timing of 12 hour light‐12 hour dark (LD) cycle is shown by alternating black and white rectangles on top of the histograms

The circadian control of timing of eclosion, similar to locomotor activity, is a critical output of circadian pathways. We sought to determine the eclosion rhythm in *GMR*>CHMP2B^Intron5^ flies. As expected, the wild type and *GMR*‐GAL4 flies preferentially eclosed around light off‐on (dawn) transition and *period* mutant (*per^0^*) did not show any preference for eclosion time in 12 hour light‐12 hour dark (LD) cycle (Figure 2, *P* < 0.01, chi‐squared test, n_wt_ = 100, n*_GMR_*_‐Gal4_ = 59, n*_per0_* = 67). In contrast, the *GMR*>CHMP2B^Intron5^ flies showed a significant alteration of the eclosion rhythm as evidenced by lack of robust peak of eclosion around dawn (Figure 2, *P* < 0.01, chi‐squared test, n = 77).

**Figure 3:**
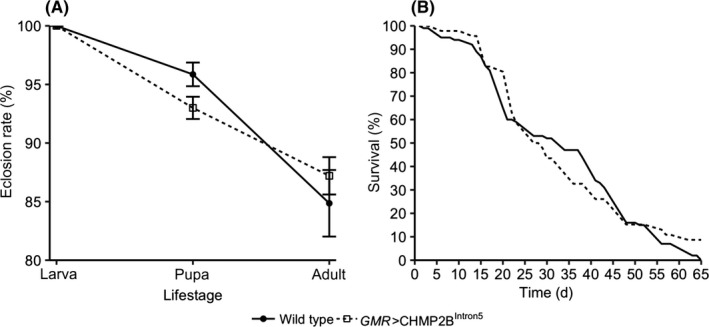
Line charts plotting the (A) development rate and (B) longevity show that the *GMR*>CHMP2B^Intron5^ flies have similar pupation and eclosion rates compared to that of wild type (*Canton‐S*) flies (wild type—n = 7 batches of 100 larvae each and *GMR*>CHMP2B^Intron5—^n = 5 batches of 100 larvae each; error bars—standard error of measurement; pupation—*P* = 0.07; eclosion—*P* = 0.49; Welch's *t* test). The *GMR*>CHMP2B^Intron5^ flies have a similar lifespan compared to that of wild type flies (wild type—n = 100 and *GMR*>CHMP2B^Intron5—^n = 46; *P* = 0.8; Wilcoxon rank test)

We then sought to determine whether *GMR*‐driven CHMP2B^Intron5^ expression has global physiological effects on flies. For this we compared the developmental rates and survival rates of wild type and *GMR*>CHMP2B^Intron5^ flies. The pupation, eclosion (Figure 3A, pupation—*P* = 0.07; eclosion—*P* = 0.49, Welch's *t* test, n = 5‐7 of 100 larva each) and survival rates (Figure 3B, *P* = 0.8, Wilcoxon rank test, n = 48‐100 flies) were similar between the wild type and *GMR*>CHMP2B^Intron5^ flies suggesting that *GMR*‐driven expression of CHMP2B^Intron5^ alters the circadian rhythm without affecting the overall development and longevity of flies.

### 3.3 *GMR*‐driven CHMP2B^Intron5^ expression disrupts patterning of a subset of *tim*‐positive neurons in the optic lobe

**Figure 4:**
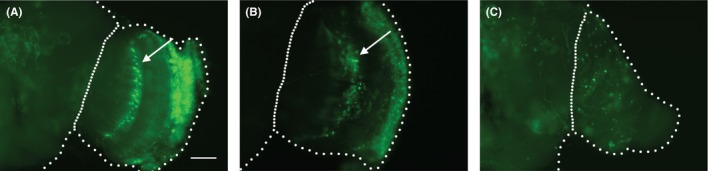
Representative images of whole mount brains of (A) *GMR*>GFP, (B) *GMR*>GFP + CHMP2B^Intron5^, and (C) *GMR*>GFP + Hid show that *GMR*‐driven expression of CHMP2B^Intron5^ causes disruption of patterning in the band of *GMR*‐positive cells in optic lobe. The band of *GMR*‐positive cells in the optic lobe and the size of optic lobe is more severely damaged in the *GMR*>GFP + Hid flies compared to that of *GMR*>GFP + CHMP2B^Intron5^ flies. Large dotted lines outline the brain while small dotted lines demarcate the optic lobe/central brain boundary. Arrows in (A) and (B) mark the band of large cells in optic lobe; scale bar: 50 μm

To examine the effect of *GMR*‐driven CHMP2B^Intron5^ expression on the central nervous system neurons, we first used *GMR*>GFP flies to determine the *GMR* expression pattern in the brain. *GMR* drives expression in a band of cells in the optic lobe (Figure 4A arrow). When we co‐expressed GFP and CHMP2B^Intron5^ using *GMR* driver, the patterning of the optic lobe cell band was significantly disrupted (*P* < 0.001, Welch's *t* test, n = 5 independent preparation for each genotype), indicating partial degeneration of those cells (Figure 4B arrow and Supplementary Figure S3). As reference, we tested if *GMR*>Hid flies showed more damage than *GMR*>CHMP2B^Intron5^ flies. Indeed, *GMR*>Hid flies showed more severe disruption in the patterning of the optic lobe cell band. Also, *GMR*>Hid + GFP flies had a much smaller optic lobe with no evidence of the band of large cells seen in the *GMR*>GFP flies (Figure 4C and Supplementary Figure S3).

**Figure 5:**
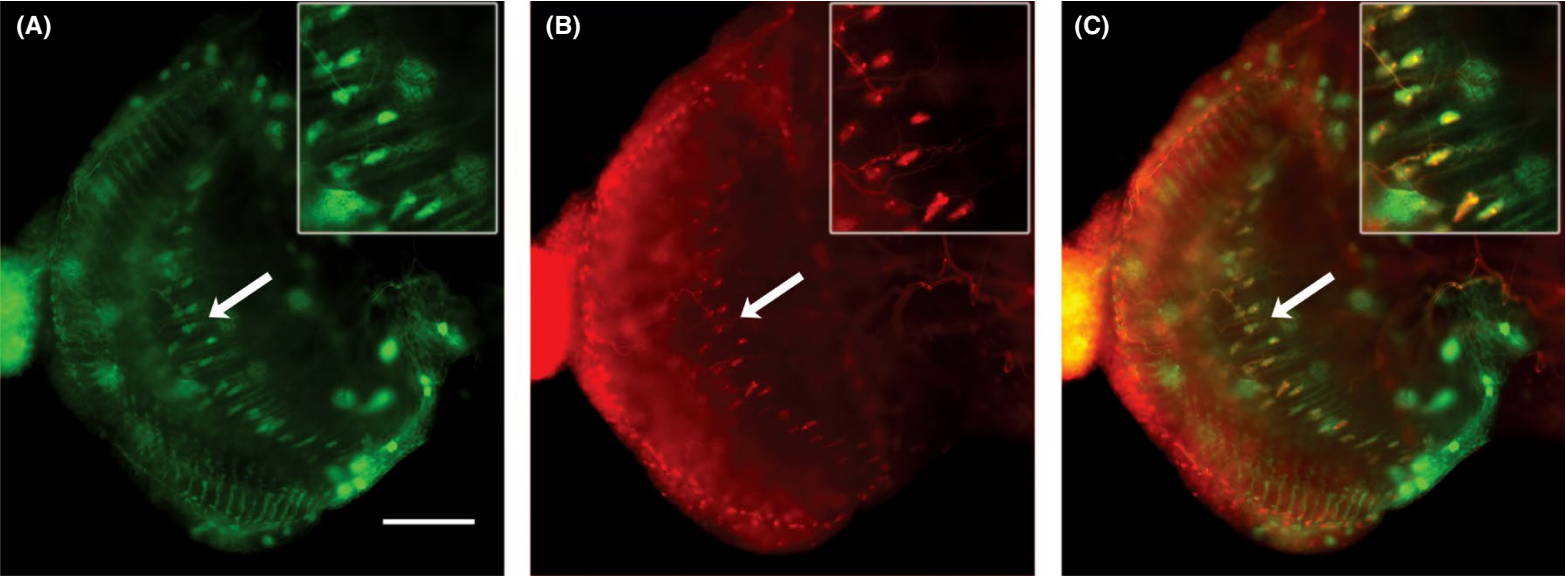
Representative images of whole mount brains of (A) *GMR*>GFP and (B) *tim*‐DsRed show that *GMR*‐driven expression of GFP in a band of cells in the optic lobe partially overlaps with *tim*‐driven expression of DsRed as shown in the (C) merged image. Arrows highlight the relevant band of cells with substantial overlap of DsRed and GFP signal, inset is a digital zoom of the area marked by arrows; scale bar: 50 μm

To determine whether the *GMR*‐positive band of cells in the optic lobe contributes to circadian rhythms, we examined whether there is an overlap between the expression pattern of *GMR* and *tim*—an important circadian rhythm regulator. We generated a new transgenic *tim* reporter line, *tim*‐DsRed, in which *tim* promoter directly controls red fluorescent protein (DsRed) expression. The *tim*‐DsRed;* GMR*>GFP flies showed a substantial overlap of DsRed and GFP signals, thereby confirming that *GMR* and *tim* partially share the expression pattern in the optic lobe (Figure 5). These data show that *GMR*‐mediated expression of CHMP2B^Intron5^ disrupts a subset of *tim* expressing cells in the optic lobe.

### 3.4 *GMR*‐driven CHMP2B^Intron5^ expression causes reduction in *tim* transcript levels

**Figure 6:**
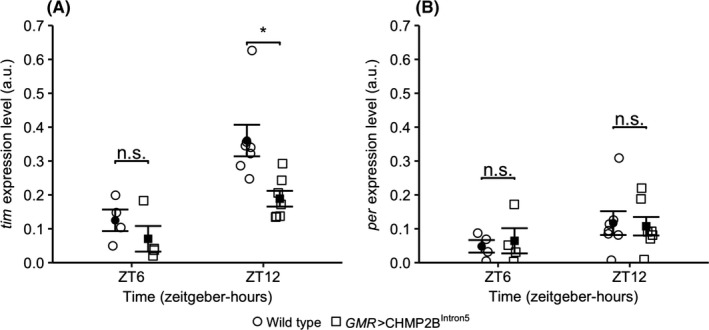
Scatter plots of the normalized transcript levels of (A) *tim* and (B) *per* at ZT6 and ZT12 show that the *GMR*>CHMP2B^Intron5^ flies have a significant reduction in *tim* transcript levels at ZT12 when compared to wild type (*Canton*‐*S*) flies (*—*P* < 0.05; Welch's *t* test; error bars: standard error of measurement; n.s.—not significant; a.u.—normalized arbitrary unit; n = 5‐7—wild type and *GMR*>CHMP2B^Intron5^). Both wild type and *GMR*>CHMP2B^Intron5^ flies show significant increase in *tim* transcript levels (ZT12:ZT6 fold change*_tim_*—wild type = 2.9; *GMR*>CHMP2B^Intron5^ = 2.6 (two‐way ANOVA for time—*P* = 0.0003 and genotype—*P* = 0.003). However, the *per* transcript levels show a trend for increase between ZT6 and ZT12 in wild type and *GMR*>CHMP2B^Intron5^ flies (ZT12:ZT6 fold change*_per_*—wild type = 2.3; *GMR*>CHMP2B^Intron5^ = 1.6; two‐way ANOVA for time—*P* = 0.11 and genotype—*P* = 1.0)

The molecular basis of circadian rhythms relies on precise cyclic fluctuations of transcriptional activation and post‐translation degradation of core circadian pacemaker genes such as *tim* and *per*. The *per* and *tim* transcript levels are lowest around dawn (ZT0), accumulate during the day, and peak in the evening (ZT12) in 12 hour light‐12 hour dark (LD) cycle.^28^, ^29^ We quantified *tim* and *per* transcript levels in the *GMR*>CHMP2B^Intron5^ flies raised in 12 hour light‐12 hour dark (LD) cycle. The *GMR*>CHMP2B^Intron5^ flies showed a significant reduction in *tim* transcript levels at ZT12 (1.9 fold, *P* < 0.001, Welch's *t* test, n = 5‐7) but not at ZT6 (*P* = 0.31, Welch's t test, n = 5‐7) and not in *per* transcript levels at both ZT12 and ZT6 (*P* = 0.8, *P* = 0.7 respectively, Welch's *t* test, n = 5‐7) when compared to wild type flies (Figure 6). Both wild type and *GMR*>CHMP2B^Intron5^ flies showed a significant increase in *tim* transcript levels (ZT12:ZT6 fold change*_tim_*—wild type = 2.9; *GMR*>CHMP2B^Intron5^ = 2.6; two‐way ANOVA for time—*P* = 0.0003 and genotype—*P* = 0.003). These data show that *GMR*‐mediated expression of CHMP2B^Intron5^ causes a deficit in the accumulation of *tim* transcripts during the lights on phase of the 12 hour light‐12 hour dark (LD) cycle.

## 4 DISCUSSION

CHMP2B^Intron5^, a gain‐of‐function mutant CHMP2B variant, is associated with chromosome‐3 linked FTD (FTD‐3). CHMP2B^Intron5^ alters the composition of ESCRT‐III complex, which prevents its dissociation and disrupts proper formation of MVBs in humans, mice, and flies.^10^, ^30^, ^31^, ^32^ As a result, CHMP2B^Intron5^ causes accumulation of malformed endosomes and autophagosomes in FTD‐3 patient fibroblasts and neurons,^33^ mice,^30^, ^34^ flies,^31^ and neuroblastomas.^35^ At the molecular level, CHMP2B^Intron5^ causes misregulation of cell‐surface receptor turnover thereby resulting in upregulation of their signaling pathway.^10^, ^11^ Previously, we showed that ectopic expression of CHMP2B^Intron5^ using *GMR*‐GAL4 driver in *Drosophila* causes melanization in the retinal photoreceptors associated with upregulation of Toll pathway activity.^10^ Since CHMP2B is associated with non‐specific regulation of protein turnover, we wondered what other pathways are misregulated in *GMR*>CHMP2B^Intron5^ flies. In this study, we demonstrate at the behavioral, cellular, and molecular level that *GMR*>CHMP2B^Intron5^ flies also exhibited disrupted circadian locomotor rhythms.

At the behavioral level, *GMR*>CHMP2B^Intron5^ flies showed reduced free‐running locomotor activity period. Whereas entrainment to light appears essentially normal, the rhythmicity of the clock is affected, as evidenced by the shortened free‐running period (Figure 1). The *GMR*>CHMP2B^Intron5^ flies also showed mild deficit in eclosion rhythms (Figure 2). These data show that CHMP2B^Intron5^ causes moderate disruption of circadian rhythms. Given comparable developmental and mortality rates between wild type and *GMR*>CHMP2B^Intron5^ flies (Figure 3), the *GMR*>CHMP2B^Intron5^ flies appear just as robust as wild type flies in development and survivorship. Therefore, the CHMP2B^Intron5^ mediated circadian rhythm phenotype does not appear to be a side effect of other global physiological defects.

Current understanding of the cellular basis of circadian locomotor rhythms describes a complex neuronal circuit that connects light sensitive structures (eg photoreceptors cells in retina, ocelli, and H‐B eyelet) to about 150 circadian pacemaker neurons arranged in multiple clusters in the fly brain. Previously, we have described degeneration of photoreceptor neurons in the retina of *GMR*>CHMP2B^Intron5^ flies.^10^ The ablation of photoreceptor cells causes defects in entrainment to low intensity light but the free‐running rhythm is maintained.^36^ However, in *GMR*>CHMP2B^Intron5^ flies the most obvious phenotype is shortening of the free‐running locomotor period and the light entrainment is essentially unaffected thereby indicating involvement of additional cells in the circadian phenotype (Figure 1). Therefore, we determined if *GMR*‐GAL4 drove expression in any regions in the brain that might be associated with free‐running locomotor circadian rhythms. We did not observe *GMR*‐driven expression in any putative clusters of circadian pacemaker neurons (not shown). However, *GMR*‐GAL4 does drive expression in a band of cells in the optic lobe and a substantial portion of these cells are also *tim*‐positive (Figure 5). Further, these *GMR*‐positive neurons do not appear to have *cry*‐, *per*‐, and *clock*‐driven expression (data not shown; unpublished data). The l‐LN_v_s send projections into the optic lobe^14^ and may possibly innervate the *GMR*‐positive neurons in the optic lobe. Therefore, it appears that the disruption of the patterning of the *GMR*‐ and *tim*‐positive neurons in the optic lobe in *GMR*>CHMP2B^Intron5^ flies (Figure 4) is likely contributing to the circadian locomotor rhythm phenotype (Figure 1). We find these results significant because they demonstrate the ability of CHMP2B^Intron5^ to generate circadian rhythm defects and identify a subset of neurons in the optic lobe with overlapping expression driven by *GMR* and *tim* drivers.

A mild disruption in eclosion rhythms was also observed in *GMR*>CHMP2B^Intron5^ flies. An essential component for establishing eclosion rhythms in flies is Tim‐dependent oscillations in the prothoracic gland peripheral clock.^21^ According to Myers et al, light input from peripheral clock cells likely contributes in cell autonomous Tim oscillations in prothoracic gland to generate eclosion rhythms. It is tempting to speculate that *GMR*‐GAL4‐driven expression of CHMP2B^Intron5^ in photoreceptor cells may disrupt the light input into prothoracic gland thereby resulting in defects in eclosion rhythms. We cannot rule out the possibility that additional *GMR*‐positive cells that are *tim*‐negative may contribute to the eclosion and locomotor circadian phenotypes.

As a biochemical measure of circadian rhythms, we observed transcript levels of *tim*, which is one of the key components of the molecular circadian clock. In *GMR*>CHMP2B^Intron5^ flies, *tim* transcript appears to cycle normally as evidenced by significantly higher levels at ZT12 compared to ZT6 time point (Figure 6). However, *tim* transcript level was significantly lower at ZT12 in *GMR*>CHMP2B^Intron5^ flies compared to wild type controls (Figure 6). Analysis of *tim* transcript provides robust assessment of the molecular framework of the circadian rhythm in flies and mammals.^37^ In wild type flies, *tim* exhibits a robust circadian oscillation during which the highest transcript levels are recorded in early evening (ZT12) and the highest protein levels at night (ZT18‐20).^38^ The transcription and translation of *tim* and *per* are regulated by autoregulatory negative feedback loops. Accordingly, accumulation of Tim‐Per heterodimers directly inhibits Cyc‐Clk‐mediated transcription of *tim* and *per* genes. Activation of Cry by light in morning initiates Tim degradation thereby breaking Tim‐Per heterodimers and subsequent degradation of Per monomers. The reduction in Tim and Per during daylight triggers the transcription of *tim* and *per* and begins the circadian cycle anew.^16^ Therefore, a biochemical deficit in *tim* expression is expected to propagate into a physiological defect in circadian pacemaker cells and/or peripheral clock cells.

Our findings have also contributed to further characterization of the expression profile of *GMR*‐GAL4 driver, which is primarily used to induce ectopic expression of transgenes in the *Drosophila* eye. While there have been reports that suggest *GMR*‐GAL4 has a broad expression in cells outside of retina including the central nervous system,^23^ we showed that the *GMR*‐GAL4 expression profile includes cells important to the circadian rhythms. The most remarkable novel region of the *GMR* expression pattern in the central nervous system is a band of cells in the optic lobe. This evidence further establishes that *GMR* drives expression in cells that are critical to the normal function of circadian locomotor and eclosion rhythms.

Our data indicate that CHMP2B^Intron5^ causes a relatively mild phenotype compared to the expression of apoptosis effector Hid (Supplementary Figure S2). Therefore, it appears that CHMP2B is the not the primary regulator of turnover of circadian proteins, which are primarily regulated by proteasome‐mediated degradation.^39^ How does mutant CHMP2B causes disruption in circadian rhythms? There are at least two non‐mutually exclusive possibilities to explain the role of CHMP2B/ESCRT in regulation of circadian pacemaker proteins. First, CHMP2B and/or ESCRT pathway directly modulates homeostasis of circadian rhythm proteins. Recently, numerous studies have shown a connection between circadian rhythms and endocytosis and autophagy. Mutations in CHMP2B and other ESCRT components are associated with accumulation of ubiquitinated proteins in abnormal autophagosomes.^31^, ^40^, ^41^ The autophagy pathway was shown to play a modulatory role in degradation of CRY1 and BMAL1 (mammalian homolog of Cycle) during circadian regulation of glucose metabolism in mice liver.^42^, ^43^ The autophagy pathway and TOR pathway (a negative regulator of autophagy) were shown to regulate circadian locomotor rhythms.^44^ Ectopic expression of mutant Shibire (*Drosophila* homolog of Dynamin)—an ATPase required for scission of plasma membrane to form endocytic vesicles, causes disruption of circadian rhythm.^45^

Second, mutations in CHMP2B/ESCRTs disrupt the physiology of circadian neurons due to misregulation of non‐circadian proteins thereby indirectly causing circadian rhythm phenotypes. This notion is supported by findings in the *Drosophila* model of Fragile X syndrome (FXS), where loss of *Drosophila* Fragile X Mental Retardation Protein (dFMRP) causes circadian rhythm deficits because of defective synaptic plasticity of circadian neurons.^46^, ^47^ Interestingly, dFMRP is a negative regulator of Shrub—an ESCRT‐III component. Therefore, circadian deficits in the FXS fly model due to loss of dFMRP can be attributed to endosomal‐lysosomal defects due to Shrub gain‐of‐function. Indeed, we have previously reported that *GMR*‐driven overexpression of mouse homolog of Shrub (*GMR*>mSnf7‐2) causes melanization and retinal degeneration phenotype similar to *GMR*>CHMP2B^Intron5^ flies. Further studies will determine the relative contribution of these possibilities to elucidate the role of CHMP2B/ESCRTs in the circadian rhythm.

## CONFLICT OF INTEREST

The authors declare no conflict of interest.

## AUTHOR CONTRIBUTIONS

D. Lee, X. Zheng, J. Kavaler, and S. T. Ahmad designed research; D. Lee, X. Zheng, and J. Kavaler performed most of the experiments; K. Shigemori, J. B. Liu, and C. Tang contributed in the real time PCR, circadian locomotor rhythms, and in situ fluorescence experiments, respectively; D. Lee, X. Zheng, C. Krasniak, J. Kavaler, and S. T. Ahmad analyzed data; D. Lee, X. Zheng, J. Kavaler, and S. T. Ahmad wrote the manuscript.
