# Supplementary figures and images for: Expression of mutant CHMP2B linked to neurodegeneration in humans disrupts circadian rhythms in Drosophila
Source: FASEB Bioadv. 2019 Jul 11;1(8):511–20. doi: 10.1096/fba.2019-00042 (PMC6996329; doi:10.1096/fba.2019-00042)

Figure S1.

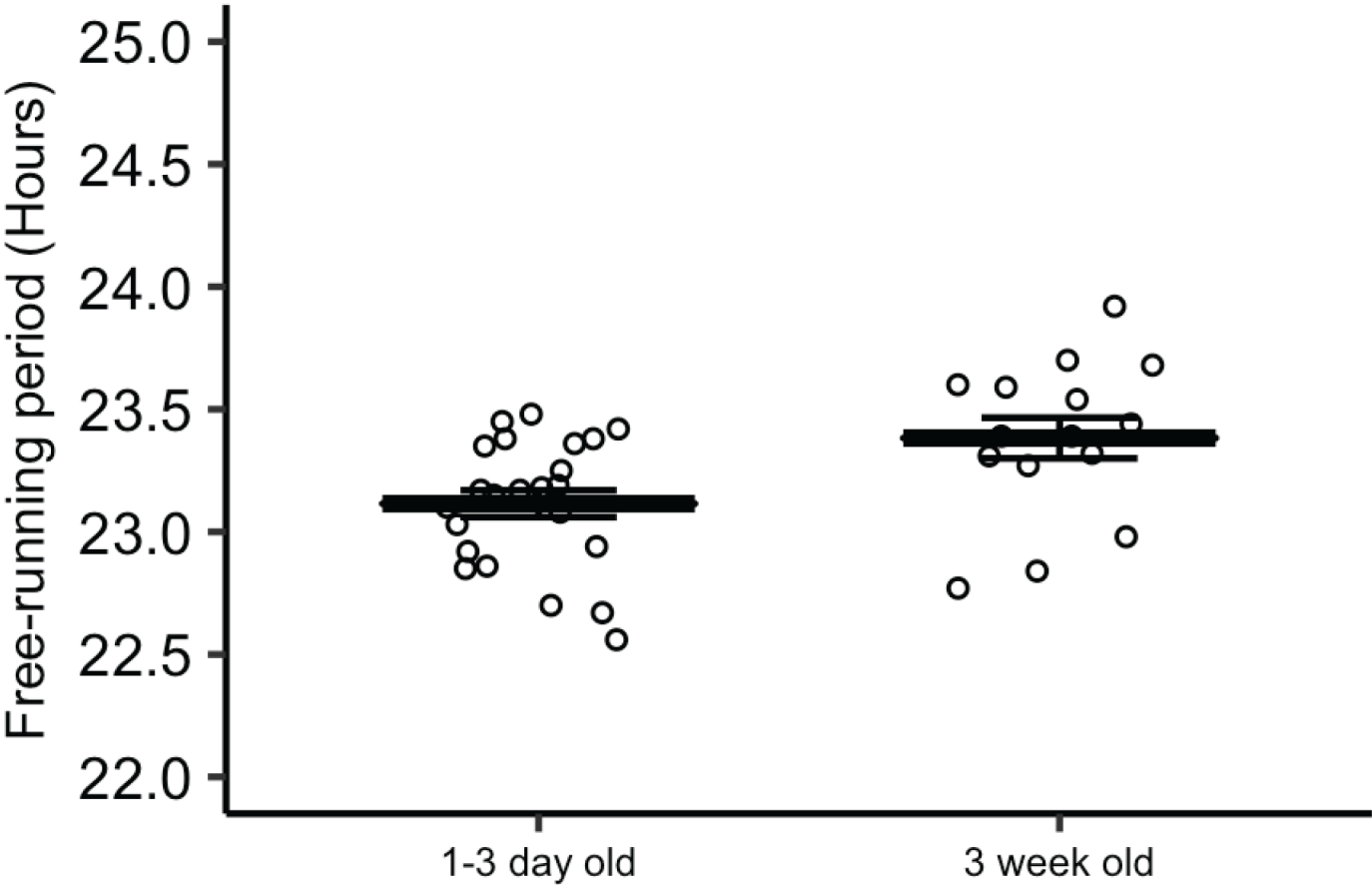

Supplement: Supplementary file 1 [file FBA2-1-511-s001.pdf]

Figure S2.

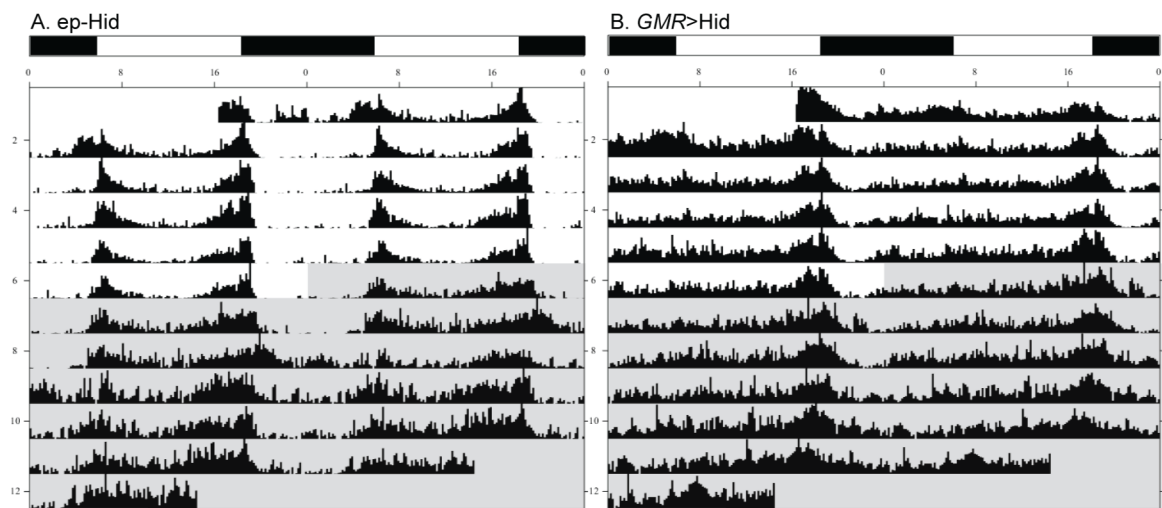

Supplement: Supplementary file 2 [file FBA2-1-511-s002.pdf]

Figure S3.

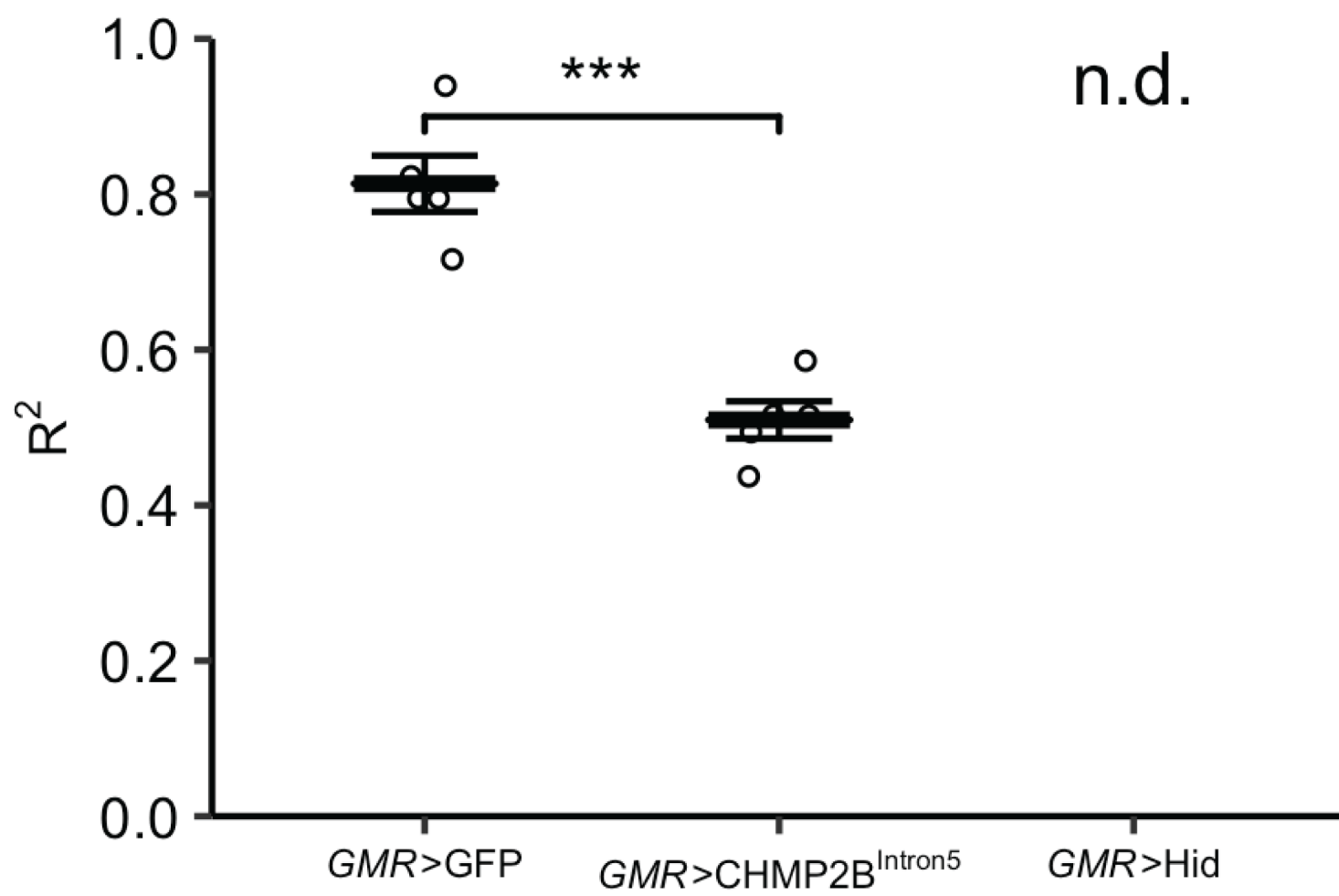

Supplement: Supplementary file 3 [file FBA2-1-511-s003.pdf]
